# PCR detection of *Campylobacter fetus* subspecies *venerealis* in smegma samples collected from dairy cattle in Fars, Iran

**Published:** 2013

**Authors:** Saeid Hosseinzadeh, Mojtaba Kafi, Mostafa Pour-Teimouri

**Affiliations:** 1 *Department of Food Hygiene and Public Health, School of Veterinary Medicine, Shiraz University, Shiraz, Iran;*; 2 *Department of Clinical Sciences, School of Veterinary Medicine, Shiraz University, Shiraz, Iran.*

**Keywords:** *Campylobacter fetus *subsp*. venerealis;* Dairy cattle, Iran, PCR

## Abstract

Bovine venereal campylobacteriosis, caused by *Campylobacter fetus* subsp. *venerealis (Cfv), *is regarded as one of the major threats to the cattle industry around the world. Abortion and infertility are two important reproductive problems in cows infected with *C. fetus* subsp. *venerealis.* Reports on the presence of *Cfv *are scarce in the cattle, in Iran. Therefore, the present study was designed to examine the presence of* Cfv *in the reproductive tract of dairy cattle either slaughtered in Shiraz abattoir or dairy herds with a history of infertility and abortion, and further to identify and differentiate this micro-organism in dairy cattle in Fars, south of Iran. A total of 95 smegma samples from the preputial cavity and the fornix of the cervical opening were collected using scraping method from bulls (n = 34) and cows (n = 61) in addition to eight samples of commercially bull frozen semen. Smegma samples were then cultured for isolation of *Cfv *and then the extracted DNA was examined for the presence of *Cfv *using an optimized multiplex PCR assay. None of the frozen semen samples examined were positive for *Cfv. *However, out of 95 smegma samples, thirteen animals (12.6%) were found positive for *Cfv *consisting of 3 males and 10 females. In conclusion, the results of the current study clearly confirmed the presence of *Cfv *using PCR in the slaughtered cattle and dairy farms with a history of poor fertility and abortion in Fars, Iran.

## Introduction

Bovine venereal campylobacteriosis is considered as one of the major threats to cattle industry around the world. The genus Campylobacter contains several important humans and animals’ pathogens affecting mainly the gastro-intestinal and reproductive tracts.^1^ *Campylobacter fetus *is divided into two major subspecies including *venerealis* and *fetus*. The subspecies *venerealis (Cfv)* is the most important cause of bovine infertility which spreads through natural mating. Although the subspecies *fetus (Cff)* can cause sporadic abortion, it is not considered to be a major cause of bovine infertility.^2^ Despite the widespread application of the artificial insemination for the management of bovine reproduction, the natural mating is still the main method of breeding in some dairy as well as beef herds. Continuous epizootic presence of venereal campylobacteriosis has been reported from countries such as Australia,^3^ the Netherlands and Belgium,^4^ Brazil,^5^ in addition to the reports regarding the re-appearance of the disease after a long period of apparent absence in countries such as the UK.^6^^,^^7^ In Iran, artificial insemination has been widely used as the preferred method in breeding programs in the last few decades in dairy cattle. However, natural mating is still the main or at least an alternative method of choice for breeding purposes in many small dairy herds under Iranian rural conditions as well as beef producing herds. In the meantime, there are reports from Iran showing cows and dairy and beef herds with the history of a transient infertility, repeat breeding syndrome and abortions.^8^^,^^9^ According to the latest information published by the International Office of Epizootics (OIE), the disease has been confirmed but no clinical aspects of the disease have been attended in Iran.^10^

The subspecies* venerealis *is an extra-cellular, motile, gram-negative, microaerophilic rod bacterium. Infected bulls may carry the micro-organism in the preputial cavity indefinitely. The non-immune female, after coitus with the infected bull, becomes infected in most occasions and the micro-organism multiply in the vagina and then spreads into the uterus causing a mild, sub-acute mucopurulent endometritis. Because of the difficulties in isolating *C. fetus*, the laboratory diagnosis of the infection is always problematic.^11^^,^^12^ Detecting the micro-organism using feasible and reliable molecular approaches are the key factors in preventing the infection.^3^^,^^13^ Hum *et al*. and Shulze *et al*. could potentially solve the difficulties of culture and of cross-reactivity between *Cfv* and *Cff* using PCR methods. Recently in the South Africa^14^ a PCR method with MG3F and MG4R primers was able to show a sensitivity and specificity of 85.7% and 99.0%, respectively in samples scraped from preputial cavity. Different serological techniques were also previously employed in order to identify the micro-organism. For example, Mshelia *et al.* were able to detect 11.0% positive cases in the vaginal mucus using an enzyme-linked immune sorbent assay (ELISA) for the detection of species-specific IgA antibodies.^16^ Moreover, Figueiredo *et al*. employed a direct fluorescent antibody test (DFA), showed the sensitivity and specificity of 92.5% and 88.8%, respectively.^16^ Among different laboratory methods available for diagnosis of BVC, the molecular investigative techniques seem an excellent choice for the epidemiological and diagnostic studies.^12^

Despite natural mating in cattle under rural conditions in Iran and the occurrence of the related clinical signs of venereal campylobacteriosis, reports on the presence of *Cfv *are scarce in cattle in Iran. Thus, the following study was designed to investigate the presence of *Cfv *in dairy cattle in Fars-Iran*. *In order to overcome the shortcomings associated with previous conventional laboratory techniques, a PCR assay was optimized and applied into our work to evaluate the molecular assay for detection of *Cfv*.

## Materials and Methods

**Sample collection. **A total of 95 smegma samples were collected from the cows (n = 22) and bulls (n = 20) slaughtered in Shiraz abattoir in addition to the cows (n = 39) and bulls (n = 14) of dairy herds with a history of abortion and infertility. The Shiraz abattoir is a place that cattle either healthy or with reproductive problems such as infertility and with a history of abortion are routinely slaughtered. Therefore, there were bulls and cows in the abattoir hailing from a herd using natural mating or artificial insemination. All bulls sampled had more than three years of age in the present study. The dairy herds (n = 11) in which the samples were obtained were routinely using artificial insemination as well as natural mating in their breeding programs. In addition, samples from eight frozen semen processed by three different AI Iranian organizations were screened for the presence of *Cfv*. The tip of a bovine uterine pipette was scratched in different directions and then was used as the device for collecting the smegma. Prior to each sampling, hair of the prepuce was cut and the area was cleaned using normal saline. The smegma samples were collected from preputial cavity in bulls and the fornix around the cervical opening in cows using scraping method as previously described.^17^ The collected smegma samples were instantly kept in the tryptic soy broth (TSB; Merck, Darmstadt, Germany) as a transport medium. Then, samples were submitted to the laboratory within 1 to 2 hr. The TSB was supplemented with 10 mg L^-1^ of amphotricin B, rifampicin, trimethoprim, vancomycin and ceftriaxon (Merck, Darmstadt, Germany) as previously described.^18^

**Sample enrichment. **The samples were then cultured under microaerophilic conditions enriched at 42 ˚C for 2 to 3 hr before being further incubated at 37 ˚C for 44 hr.^20^ One milliliter of the enriched samples were used to extract the chromosomal DNA. 

**DNA** **extraction. **Total genomic DNA was extracted using phenol-chloroform isoamyl alcohol (Merck, Darmstadt, Germany). The specimens were pelleted followed by adding 250 µL buffer I and buffer II containing RNase (CinnaGen, Tehran, Iran). A 550 µL volume of phenol was added to the aliquots before being centrifuged at 10000 *g* for 5 min, the supernatant clear phase was then collected into new eppendorf tube and the latter stages was repeated twice in order to wash cell derbies. A 0.1 volume of sodium acetate 0.1 M was then added to the tubes, washed twice using ethanol 100 and 80%, respectively. The tubes were then centrifuged at 12000 *g* for 15 min. The pellet was finally dried and re-suspended in 30 µL Tris-EDTA buffer, kept at -20 ˚C until further use.

**PCR amplification. **One species/strain specific sets of primers, that amplify the partial region of mitochondrial ND1 (NADH dehydrogenase subunit 1; CinnaGen, Tehran, Iran) gene, were used to detect the *Cfv*.

**Fig. 1:**
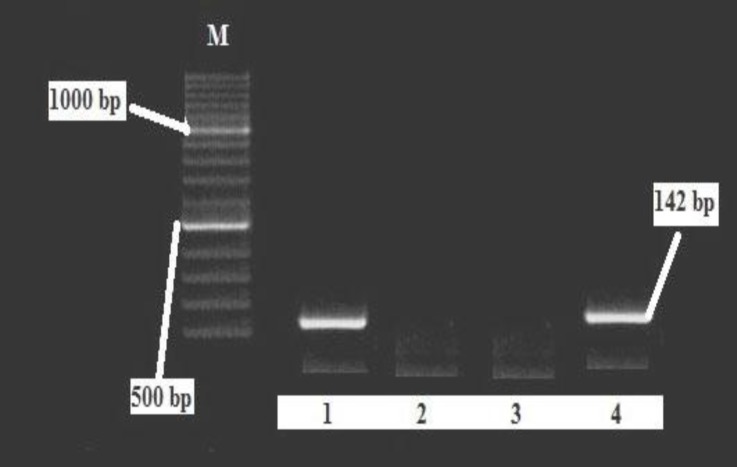
Gel electrophoresis of PCR products using primers on DNA of some isolates and positive control. Lane 1: Positive control (142 bp), Lane 2: Negative control (no template), Lane 3: Negative samples Lane 4: positive sample (142 bp), Lane M: 100 bp ladder (Fermentas).

PCR was carried out on 3 μL of DNA template in a final reaction mixture of 25 μL containing 2.5 μL of 10X PCR buffer, 3mM MgCl_2_, 200mM of each of dNTPs (CinnaGen, Tehran, Iran), 400µM of each forward and reverse primer, 2 units of Taq DNA polymerase (CinnaGen, Tehran, Iran). The PCR cycling was performed in a gradient thermocycler (Eppendorf, Hamburg, Germany) with an initial denaturation step of 94 ˚C for 10 min. followed by 30 cycles of 95 ˚C for 20 sec, 50˚C for 20 sec and 72 ˚C for 2 min. The PCR was terminated with a final extension step at 72 ˚C for 10 min.^3^ PCR products were electrophoresed on a 1.5% ethidium bromide-stained agarose gel (Fig. 1), (CinnaGen, Tehran, Iran) and the specific DNA fragments with desired size of the samples were purified and sequenced (Macro-gene, Seoul, South Korea). To analyze the sequencing data, BLASTn comparison was performed with the NCBI/ Gene-Bank database. The presence of *Cfv *was also examined in the frozen semen samples using the PCR technique as described above.

## Results

**PCR assay. **Altogether, out of 95 smegma samples, 13 (12.6%) were found positive for the presence of *Cfv *consisting of 3 males and 10 females. None of the frozen semen samples examined were positive for *Cfv. *BLASTn comparison of the sequences of 5 samples from *Cfv*-PCR against the nucleotide database did return a significant result to *Cfv* strain ATCC 19438 amino acid ABC transporter and amino acid ABC transporter permease genes.

**Table 1:** PCR results for detection of *Cfv* from smegma samples.

	**Positive (%)**	**Negative (%)**	**Total**
**Bulls**	3 (8.8)	31 (81.2)	34
**Cows**	10 (16.4)	51 (83.6)	61
**Total**	13 (12.6)	82 (81.4)	95

## Discussion

For the first time using PCR, we detected *Cfv* in smegma samples collected from the reproductive tract of cattle in Iran. The result of a previous study showed that 3.9 % of aborted fetal tissues from dairy cows were positive for *Cfv* in Tabriz, Iran.^17^ Results of the present study and those of Hamali’s *et al*. confirmed that *Cfv *is probably responsible for poor fertility, repeat breeding syndrome and abortion in some dairy farms in Iran.^8^^,^^9^ The positive diagnosis of *Cfv *in the smegma sample shad been long expected as natural mating is an alternative to artificial insemination which is routinely used in cows under Iranian dairy farms conditions. The prepuce of the bulls is the natural reservoir of the infection for naive cows through natural mating.^1^ Considering the important role of the general and reproductive health of a bull on the production performance of a dairy herd, the infection rate of 8.8% found in bulls in the present study could be an alarming call for Iranian dairy industries. The transmission rate from infected bulls to susceptible cows may approach even 100%.^19^

The aim of the present study was to merely investigate the presence of *Cfv *in the dairy cattle in Fars, Iran, and therefore our findings could not be interpreted with epidemiological viewpoints. However, an overall 12.6 % positive for *Cfv *in the present study are, regardless of the diagnostic techniques, comparable to the infection rates of 8.2, 22.0, 21.0 and 18.0% in Czech Republic,^10^ Argentina,^20^ Japan,^21^ and New Zealand,^22^ respectively. More researches are needed to study specifically the epidemiology and risk factors associated with bovine venereal campylobacteriosis in different regions of Iran.

Results of the present study showed that none of the frozen semen samples were positive for *Cfv*. This implies the existence of the standard biosecurity measures in semen processing and therefore, allows us to recommend the extensive use of artificial insemination in dairy farm in Fars, Iran. Transmission of *Cfv *could also occur during artificial insemination with infected semen or through contaminated insemination equipment.^23^^,^^24^

No samples were found positive when we tried to culture the smegma samples using the selective culture conditions in the present study. Campylobacter spp. are not cultured and isolated easily,^11^ because they are fastidious in nature and have low survival rates with delicate requirements for growth in media. This could explain the under estimation of the true infection rate for bovine venereal campylobacteriosis when culturing approach is solely used.^1^ It is essential to consider that preputial and cervico-vaginal scraped specimens contaminated with urine, feces and semen may potentially interfere with the direct culture and/or ordinary DNA extraction techniques.^25^^,^^26^ In addition, the choice and availability of media is very critical to the successful culture of campylobacter from animal samples.

In conclusion, the results of this study clearly confirm the presence of *Cfv *in slaughtered cattle and dairy farms with a history of poor fertility and abortion in Fars, Iran.
